# Circulating Levels of IL-8 and MCP-1 in Healthy Adults: Changes after an Acute Aerobic Exercise and Association with Body Composition and Energy Metabolism

**DOI:** 10.3390/ijms241914725

**Published:** 2023-09-29

**Authors:** Rudite Lagzdina, Maija Rumaka, Gita Gersone, Peteris Tretjakovs

**Affiliations:** Department of Human Physiology and Biochemistry, Faculty of Medicine, Riga Stradiņš University, LV-1007 Riga, Latvia; rudite.lagzdina@rsu.lv (R.L.);

**Keywords:** IL-8, MCP-1, aerobic exercise, metabolism

## Abstract

The most recent WHO recommendations about physical activity emphasise the importance of total exercise volume above the significance of the duration of each bout. This study examined whether acute aerobic exercise changes circulating levels of IL-8 and MCP-1 and if these changes are associated with body composition and energy metabolism. Healthy adult volunteers completed a 10 min walking–running exercise on a treadmill. Indirect calorimetry was used to determine their resting metabolic rate (RMR) and energy expenditure (EE) during the exercise. Pre-exercise levels of IL-8 and MCP-1 were similar in both sexes. There were positive correlations of pre-exercise IL-8 with body mass, waist circumference, and lean body mass in men and pre-exercise MCP-1 with RMR in women. The exercise led to an increase in IL-8 of 68% and a decrease in MCP-1 of 74% of participants. An increase in post-exercise IL-8 in men was associated with greater walking EE and a greater increase in walking EE. The increase in post-exercise MCP-1 was associated with a lower RMR and running EE in women. There are both sex and individual variations in changes in chemokine secretion in response to the same exercise situation and their associations with values of metabolic parameters.

## 1. Introduction

The current evidence suggests that physical activity modulates immune reactions in the body, which enables necessary tissue response to the damage and the repair functions. The proven ability of physical activity to reduce the risks of non-communicable diseases (like heart disease, stroke, and diabetes) could be associated with the influences of physical activity on the inflammatory processes involved in the pathogenesis of these diseases [1]. Regulation of recruitment of immune cells and intracellular reactions is carried out through the signals of extracellular molecular regulators: cytokines. Interleukin-8 (IL-8) and monocyte chemoattractant protein-1 (MCP-1) are some of the oldest known and the most significant in the complexity of these signalling pathways [2].

Chemokine of the CXC subfamily IL-8 signalises through G protein-coupled receptors CXCR1 and CXCR2 in cells. The presence of the gene group that encodes IL-8 and its receptors in humans and various animals indicates the importance of chemokine throughout evolution [3]. It is responsible for the recruitment, migration, and activation of immune cells at sites of inflammation and, in modified form, can also target endothelial cells. In addition to macrophages, which are the main source, IL-8 in the circulation can also appear from skeletal muscles and, therefore, is counted as a contraction-regulated cytokine [2,4]. 

MCP-1, after its identification more than 30 years ago, was extensively studied. It is a polypeptide consisting of 76 amino acids and is classified as a chemotactic cytokine or chemokine of the CC subfamily. The effects of MCP-1 in cells are mediated through its binding to the CC chemokine receptor 2 (CCR2); therefore, the chemokine is also known under the name of the CC chemokine ligand 2 (CCL2) [5,6]. As studies to date show, the source of circulating chemokine can be a variety of MCP-1-producing cell types. Either immanently or upon different stimuli, expression of MCP-1 occurs in vascular endothelium, epithelial and brain cells, various immune cells, and many others; however, muscle, adipose tissues, and their resident macrophages can be recognised as the most important producers of MCP-1 in the human body [4,7,8,9].

Due to their influence on immune cells, IL-8 and MCP-1 are classified as pro-inflammatory cytokines and are implicated in tumour growth and metastasis progression, atherosclerotic, cell senescence, and degenerative processes [8,10,11]. These connections between cytokines and the pathogenic mechanisms behind chronic inflammatory disorders may allow the use of their circulating levels as potential biomarkers and their signalling pathways as potential therapeutic targets [12,13]. 

Studies show that bouts of exercise elicit an acute pro-inflammatory response as a reaction needed for the repair of damaged muscle structures. Many different cytokines, including IL-8 and MCP-1, are released, and even systemic inflammatory reactions can be caused, especially after prolonged and exhaustive exercise [14]. 

The results of the studies confirm that physical training can modulate immune functions. Lifelong exercising effects against an ageing-related basal inflammatory state are not ambiguous; however, it was characterised as a beneficial factor in acute exercise-triggered pro-inflammatory response in master athletes, including the lower expression of IL-8 in muscles [15,16]. The observation that physical exercise in healthy adults reduces the surface expression of CCR2 in the intermediate monocyte subset suggests that reaction to exercise can vary from pro- to anti-inflammatory, depending on several groups of factors like type, duration and intensity of exercise, body temperature, cellular energy metabolism, level of stress, and sex hormones [17]. Low physical activity is one of the factors that induce metabolic stress, causing upregulation of MCP-1 (CCL2) and, subsequently, impairs lipid metabolism and energy regulation in metabolically active tissue [18,19]. German researchers reported different baseline levels and acute exercise-induced changes in circulating plasma proteins and chemokines, depending on the training status. They considered the results as a long-term adaptation to regular exercise, which also allows for improved exercise performance and diminishes adverse exercise-induced inflammatory and metabolic reactions [20]. The results of a study by Dogra et al. showed that even short bouts of activity performed during prolonged sitting reduce inflammatory biomarkers [21]. Although higher engagement in physical activities was associated with a lower level of IL-8, depending on its concentration, chemokine also proved to be a modulating factor in the relationships between physical activity and levels of fatigue and depressive mood in breast cancer survivors [22]. 

The influence of MCP-1 on macrophage polarisation and its role in white adipose tissue browning in mice experiments reveal that the deficiency of MCP-1 stimulates UCP-1-mediated thermogenesis and has a potential role in the regulation of body energy balance [23]. 

According to studies that examined minute-by-minute registered data in free-living situations, children and adults participate in a lot of moderate to vigorous physical activity (MVPA) in short bursts lasting less than 10 min, accounting for almost two-thirds of the total time spent in MVPA [24,25]. 

In addition, in school and occupational routines, purposefully integrated PA activities intending to promote the PA level, work, and cognitive performance or achieve various health outcomes are, in many cases, of brief duration, approximately 10 to 15 min [26]. The most recent WHO recommendations encourage people to engage in MVPA for their health and promote the importance of total physical activity volume above the significance of the duration of each bout since additional evidence was found to support the idea that bouts of physical activity shorter than 10 min are linked to health-enhancing effects [1]. Studies have shown that PA accumulated in bouts of less than 10 min, referred to as “exercise snacks”, has an even greater impact on various health outcomes, such as blood triglycerides, glucose regulation, and resting arterial blood pressure, than continuous PA (moderate-intensity) activity lasting longer than 10 min [27,28].

In light of this, the purpose of this study is to examine the changes in circulating levels of the chemokines IL-8 and MCP-1 that occur in healthy adults after acute aerobic exercise that resembles the short bursts of physical activity that people engage in daily, as well as to further determine whether the level of chemokines is associated with body composition parameters and energy metabolism.

## 2. Results

Anthropometric parameters (Table 1) of study participants stratified by sex show higher values of height, body mass, waist circumference, lean body mass, and LBMI in men, but the percentage of body fat, as predicted, was higher in women. The mean results of age and BMI were slightly higher in men.

The concentrations of both chemokines in the blood samples before (pre-exercise) and after (post-exercise) physical exercise are presented separately in male and female participants, but their values did not differ statistically significantly between the sexes.

**Figure 1:**
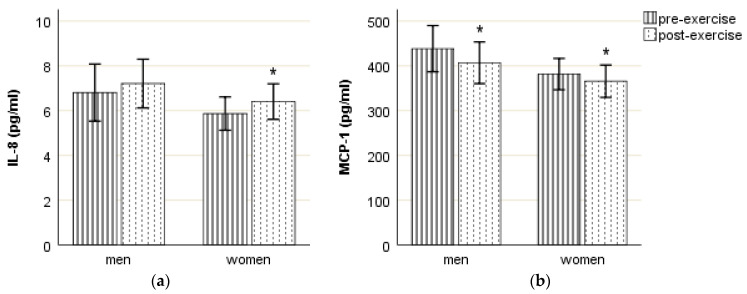
Mean (95% CI) pre- and post-exercise plasma concentration of IL-8 (**a**) and MCP-1 (**b**). * *p* < 0.05 vs. pre-exercise value of the corresponding sex.

The mean post-exercise results show a slight increase in IL-8 (statistically significant only in women) and a decrease of MCP-1 compared to the concentration of respective chemokine in the pre-exercise blood sample (Figure 1). However, the direction of the changes was not the same in all participants: IL-8 concentration in the post-exercise sample was higher than in the pre-exercise in 49 or 68% of the participants (20 men (61%), 29 women (74%)), and the concentration of MCP-1 was lower than in the pre-exercise sample in 53 or 74% of the participants (25 men (76%), 28 women (72%)).

Although IL-8 was significantly positively related to body mass, waist circumference, lean body mass, and LBMI in men, RMR was the only variable with a statistically significant positive correlation with MCP-1 in women (see Table 2). None of the assessed anthropometric or metabolic parameters significantly correlated with the pre-exercise levels of IL-8 in females or of MCP-1 in males.

**Figure 2:**
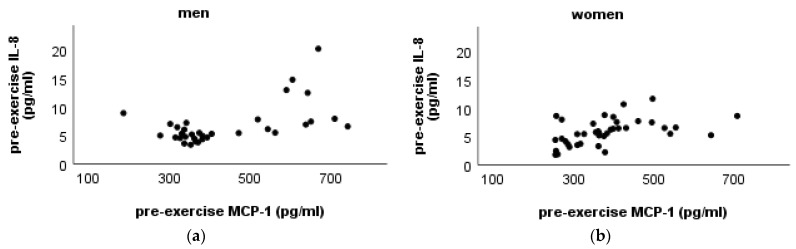
Association between concentration of pre-exercise MCP-1 and pre-exercise IL-8 in men (**a**) and women (**b**).

The association between the pre-exercise MCP-1 and IL-8 in both sexes is shown in Figure 2.

The results of Spearman’s correlation test show a positive, statistically significant correlation between the pre-exercise concentrations of IL-8 and MCP-1 in men (rho = 0.456, *p* = 0.008) and women (rho = 0.564, *p* < 0.001).

**Figure 3:**
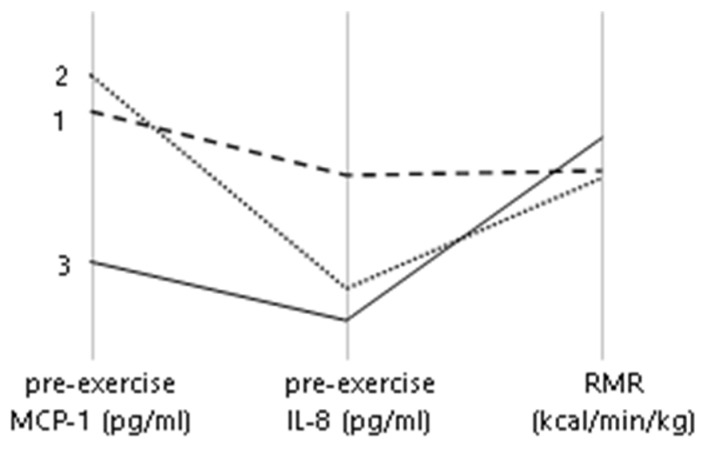
Mean results of pre-exercise MCP-1, IL-8, and RMR in clusters in men.

The distribution of results of pre-exercise concentration in men made us assume that the relationship between the concentrations of both chemokines is nonlinear; therefore, we applied cluster analysis for the results of pre-exercise IL-8 and MCP-1 and several parameters of metabolism to find possible subgroups in participants of each sex. In the men’s group, model-based clustering due to the chemokine concentration and RMR led to three clusters (Figure 3). Most of the male participants (*n* = 21, cluster 3) had low pre-exercise concentrations of MCP-1 and IL-8 and the highest RMR per body mass kg value, while in clusters 1 and 2 (in each *n* = 6), the participants had different characteristics of the concentration and lower RMR than those in the cluster 3. In women, cluster analysis of MCP-1, IL-8 and RMR or other metabolic parameters did not reveal clearly distinctive subgroups.

**Figure 4:**
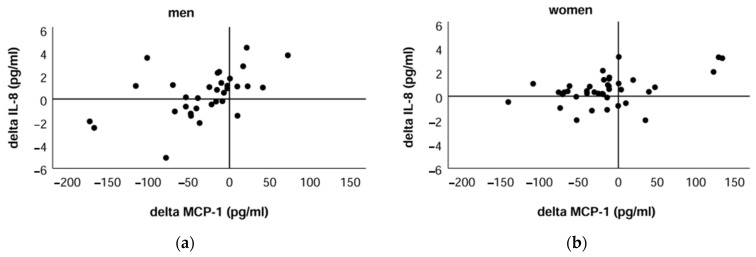
Association between IL-8 changes and changes of MCP-1 after two-stage exercise in men (**a**) and women (**b**).

Figure 4 demonstrates that in most participants, the positive delta IL-8 is associated with a negative delta MCP-1; however, other relationships between delta chemokines were also found in both men and women.

Depending on changes in the concentration of each chemokine in the post-exercise sample regarding the pre-exercise, negative and positive delta IL-8 and delta MCP-1 groups were created: as a negative delta group if the post-exercise concentration of the chemokine was lower than the pre-exercise; and as a positive delta group if the post-exercise concentration was higher. 

**Figure 5:**
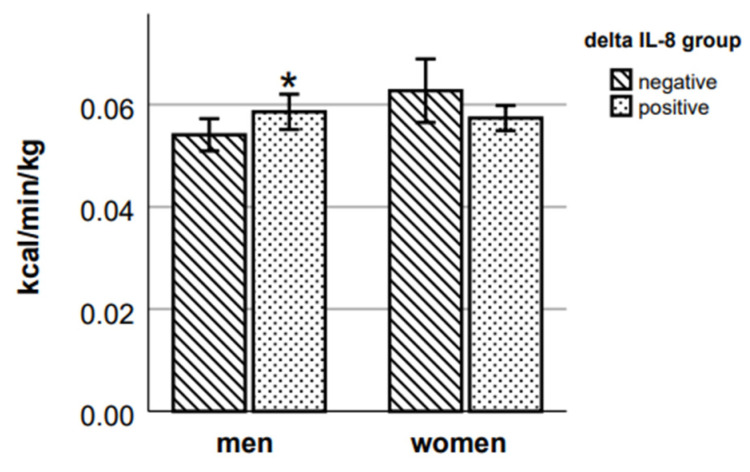
Walking EE in delta IL-8 groups. * *p* < 0.05 vs. negative group of the respective sex.

**Figure 6:**
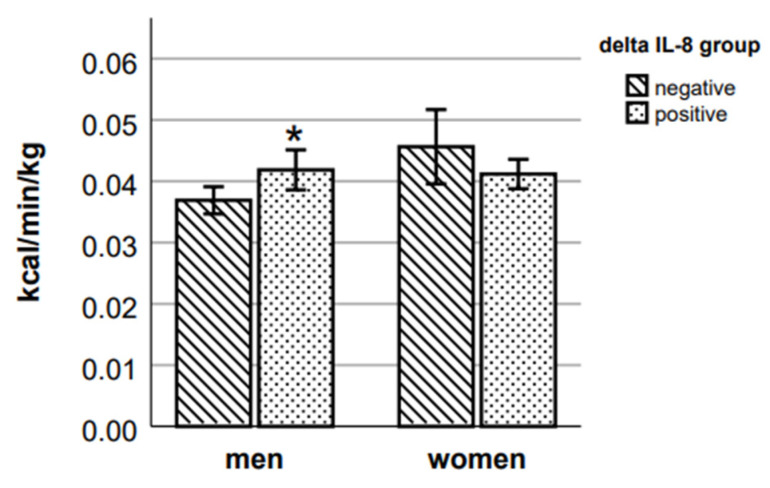
Δ walking EE in delta IL-8 groups. * *p* < 0.05 vs. negative group of the respective sex.

A comparison of energy expenditure variables between the two groups of delta IL-8 showed a statistically significant difference in walking EE (Figure 5) and Δ walking EE (Figure 6) (respective *p* = 0.036 and *p* = 0.009, Mann–Whitney test) with an 8.3% greater walking EE and 13.6% greater increase of Δ EE during walking in those male participants who had a positive delta IL-8 value. In women, the results show the opposite tendency; however, the differences in results of walking EE and Δ walking EE between delta IL-8 groups were not statistically significant.

**Figure 7:**
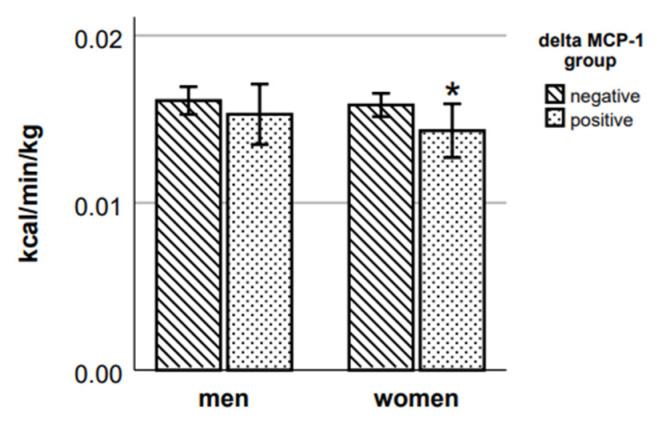
RMR in delta MCP-1 groups. * *p* < 0.05 vs. negative group of the respective sex.

**Figure 8:**
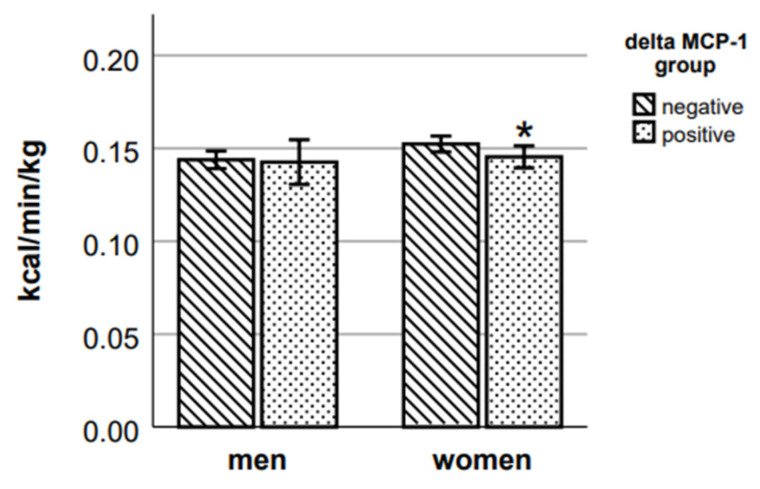
Running EE in delta MCP-1 groups. * *p* < 0.05 vs. negative group of the respective sex.

Female participants with positive delta MCP-1 had 9.8% lower RMR (Figure 7) and 4.7% lower running EE (Figure 8) (respective *p* = 0.023 and *p* = 0.039, Mann–Whitney test) than those participants whose MCP-1 decreased after the exercise. There were no statistically significant differences in RMR and in running EE between male participants of negative and positive delta MCP-1 groups.

## 3. Discussion

In this study, pre-exercise circulating chemokine levels in the blood were measured in participants under comparable circumstances, specifically at the same time of day, the time interval to prior dietary intake, and physical activity. As a result, the values may be regarded as typical baseline concentrations of IL-8 and MCP-1 in healthy adults.

We found slightly lower levels of both chemokines in women, but there was no statistically significant difference, so we were unable to speculate on the gender-dependent factors that affect the baseline level of proinflammatory cytokines. On the contrary, the study involving young athletes showed significantly higher levels of IL-8 and MCP-1 in men than in females [29]. Due to differences in other determining factors such as age, body composition, and diverse methods of determination, as well as the fact that most studies analysing chemokines and their association with physical exercise involve male participants, it is difficult to compare absolute values of chemokines between studies. The pre-exercise concentrations of IL-8 and MCP-1 recorded in our study group were comparable to values seen in other investigations with healthy middle-aged, young, untrained men and young endurance athletes with body types similar to our males [30,31,32,33]. 

However, two other studies, one on marathon runners who were anthropometrically similar to the men in our study and the other on slightly older, both male and female, lean participants, found significantly lower levels of IL-8 and MCP-1 [7,34].

The baseline circulating amounts of IL-8 and MCP-1 in healthy individuals can be predicted by the proportions of body fat and muscle mass, according to the knowledge about the cell types that produce chemokines. This notion was indirectly supported by a study that compared participant values according to body mass and BMI, where a statistically significant higher concentration was observed in the overweight–obese group compared to the lean group [35]. In a study that stratified subjects according to their training status, it was observed that endurance exercise-trained 20 to 30-year-old males had greater baseline IL-8 and MCP-1 levels than sedentary persons. Although the body composition was not specified, it is reasonable to conclude that the participants’ training status probably affected the ratio of skeletal muscle to fat mass [20].

This present study revealed that greater body mass and, particularly, lean body mass is associated with higher pre-exercise IL-8 concentration. This relationship existed and was statistically significant only in male participants. We did not observe any significant correlation between IL-8 and MCP-1 concentration and body fat amount, as would be expected according to the knowledge about fat tissue as a source of chemokines, as it was presented in the results of several studies. However, augmentation of chemokine production is mainly considered to be related to obese adipose tissue, and the reduction of body fat mass also results in a decrease of MCP-1 [18,36,37]. The results of the study revealed a moderate, positive correlation between IL-8 and waist circumference in men. This outcome allows one to assume that the relationship between the chemokine and visceral fat amount is valid also in healthy men with normal or slightly increased BMI since waist circumference is a recognised indicator of the amount of abdominal fat mass [38]. Both sexes showed a positive correlation between pre-exercise levels of IL-8 and MCP-1. These findings were predictable, given the great degree of similarity between the origins and functions of both chemokines in response to typical pro-inflammatory stimuli [16].

According to previously published data, exercise stimulates the release of adipo-myokines, and the circulating level of exercise-regulated chemokines, such as IL-8 and MCP-1, increases after the exercise session [4]. Accumulating evidence suggests that the magnitude and direction of changes in chemokine secretion in response to exercise are inconsistent. The type, duration, and intensity of exercise were found to be variables that can affect the body’s response by releasing pro-inflammatory chemokines.

Our study demonstrated the impact of acute, aerobic, and moderate to vigorous exercise on plasma levels of IL-8 and MCP-1. We discovered that the mean post-exercise IL-8 concentration was higher than the pre-exercise concentration, whereas changes in MCP-1 were the opposite, with the post-exercise concentration being lower than the pre-exercise concentration. These trends of chemokine change were common among most participants, and the mean increase in IL-8 was statistically significant only for women, while the MCP-1 decrease was statistically significant for both sexes. The cross-sectional study design and relatively homogenous study group eliminated the potential influence of alteration of body anthropometric parameters and physical fitness, which have been confirmed in several studies as determinants of variable cytokine responses at the individual level and may be relevant in longitudinal studies [15,35]. 

Dorneles et al. found that the IL-8 concentration in blood was significantly increased immediately after an acute high-intensity interval exercise session in lean and overweight–obese participants, but the increase was not any more statistically significant after a moderate-intensity interval exercise session [35].

According to Patlan et al., an increase in IL-8 concentration occurred only when the acute exercise test was carried out in normal +20 °C conditions rather than in a cold environment temperature, while Dogra et al. observed a decrease in IL-8 in a group of young and healthy adults after repeated 3 min of intense aerobic exercise, but unlike our study, both studies analysed salivary chemokine concentrations. In the last study, the post-exercise sample was collected four hours after the start of the experimental exercise session [21,39].

In most of the subjects in our study, performing acute aerobic exercise appeared to result in a decrease in post-exercise MCP-1. A drop in MCP-1 after an acute moderate exercise session was also seen in other studies in young, normal-weight participants [30,40]. Although the study did not directly examine the response of circulating MCP-1 to exercise, it was discovered that a 30-min moderate-intensity session of cycle ergometry reduced post-exercise intermediate monocyte CCR2 in healthy men and women with a high daily physical activity level [17]. This observation can be linked to the binding of circulating MCP-1 to CCR2, which promotes the internalisation of the ligand–receptor complex, resulting in the elimination of the chemokine from circulation, thus inhibiting the pro-inflammatory activation of monocytes [41]. Meanwhile, some studies have found that acute resistance and endurance exercise elevates the MCP-1 level [20,42]. The inconsistency of the findings suggests that different responses due to the type of exercise are related to chemokine functions since MCP-1 production is elevated after recovering from muscle damage in resistance exercises to maintain optimal monocyte infiltration. In his dissertation, Jajtner proposes that the extent of skeletal muscle damage caused by an exercise session, rather than the volume of activity, is a more likely explanation for the conflicting results of circulating MCP-1 alterations reported in studies using dynamic resistance exercise [33].

We attempted to find a relationship between the direction of changes in chemokines in the post-exercise sample and some characteristics of the participants; however, the results showed that the participants of the positive delta group were not statistically significantly different for either IL-8 or MCP-1 in terms of age, anthropometric indicators, or values of muscle–fat tissue composition of the participants in the respective negative delta group.

A group comparison revealed that participants in the positive and negative delta groups differed in terms of energy expenditure outcomes. Male participants with a positive delta IL-8 value exhibited a greater walking EE and greater Δ walking EE than those with a negative delta IL-8 value. The elevation of MCP-1 after exercise was related to lower RMR and running EE values in women, according to the results. A comparison of the results of the men’s subgroups in the current study indicates a certain possible relationship between the basal amount of chemokine and the intensity of metabolism, as it appeared that the cluster subgroup with lower pre-exercise IL-8 and MCP-1 concentrations was associated with the highest RMR value. Little research has been published on the relationship between chemokines and energy metabolism in humans, and these distinct associations between changes in chemokine concentration in relation to exercise and energy metabolism in our male and female participants must be considered in the context of the sexual dimorphism of the known impact of the hypothalamic–pituitary–adrenal axis on exercise-induced immune responses [29]. Our data do not provide a deeper explanation of the relationship of IL-8 change with factors that would determine energy metabolism results; however, they suggest that it exists. Jürimäe et al. also observed a strong negative correlation of the IL-8 serum level with VO_2_ max kg^−1^ in well-trained rowers and proved IL-8 as a significant predictor, alone determining 38.5% of VO_2_ max kg^−1^ variability [31]. A study on human skeletal muscle cell culture found that subjecting muscle toll-like receptors to discrete ligands increased substrate uptake and oxidation. Although these same receptors are involved in the induction of myokine secretion, including IL-8, the study did not establish a direct relationship between changes in muscle cell metabolism and myokine secretion, except for IL-6 [43].

Experiments on Ccl2 knockout mice showed that MCP-1 deficiency during 22 weeks resulted in a loss in body weight, activation of browning of subcutaneous white adipose tissue, activation of body heat generation, and an increase in oxygen consumption [23]. Although long-term changes in MCP-1 may involve different regulatory and compensatory mechanisms than acute changes, our findings suggest that MCP-1 likely takes a role in the regulation of metabolic processes in humans, too.

Emphasising the knowledge gap about gender variations in chemokine expression in blood, the current study provides results of participants of both sexes. Our findings support the notion that relationships found only in males or females cannot be generalised without additional evidence. The health status of the participants and the distribution of their body composition parameters around the normal range allowed us to interpret the pre-exercise IL-8 and MCP-1 data as normal baseline concentrations of these chemokines in healthy people. The changes in both chemokines were measured after exposure of the study participants to physical load, which, due to its intensity and duration, can resemble those physical activities that many adults experience in the context of daily life as occupational, home, or other activities. Taking into account the latest WHO recommendations on PA, this study provides insight into the influence of acute aerobic exercise on pro-inflammatory cytokine secretion. 

The one limitation of our study is the lack of a control group. The availability of IL-8 and MCP-1 values from control subjects throughout the same period would rigorously prove whether the performed PA was the main reason for the alteration in the concentration of both chemokines. Additionally, repeated measurements of chemokine concentration at different time intervals after exercise would give more information on the effect of such physical exercise on the regulation of inflammatory responses. Another limitation could be the inability to exclude the effect of variations in physical fitness among individuals and variability in their food and supplementation habits on chemokine secretion and its impact on metabolic parameters.

## 4. Materials and Methods

### 4.1. Study Subjects

Seventy-two participants (33 males and 39 females) were included in the study. Inclusion criteria consisted of being 20–50 years of age, with no acute or chronic medical conditions, and no use of any medication during the study. The study was approved by the local ethics committee of Riga Stradiņš University (decision No. 14/31.10.2013), and informed consent was obtained from each participant upon entry into the study. 

### 4.2. Body Composition and Metabolism Measurements

Study participants entered the laboratory in the morning after an overnight fast and with limited PA prior to the test to obtain more accurate findings. All measurements were carried out in the morning between the hours of 8:00 and 10:00 on the same day. The body height was measured and expressed to the nearest 1.0 cm, and a Tanita MC-180 MA multi-frequency bioimpedance analyser (Tanita Corp., Tokyo, Japan) was used to measure body mass and evaluate body composition, including fat and lean body mass (LBM). Body mass index (BMI) and lean body mass index (LBMI) as LBM (kg)/height (m)^2^ were calculated. Indirect calorimetry was used to determine the resting metabolic rate (RMR) and to measure energy metabolism during exercise tests. A gas analysis system (Oxycon Pro, Carefusion, Höchberg, Germany) was used to perform breath-by-breath respiratory gas analysis.

Following the recommendations for measuring RMR in healthy people, RMR measurements were carried out on subjects who remained supine [44]. After removing the first five minutes’ worth of data from the 30-min recording, the coefficient of variation (COV) for oxygen uptake (VO_2_) and carbon dioxide release (VCO_2_) was calculated to establish whether a steady state had been reached. The RMR was determined using the 5-min interval with the lowest COV (10%).

Participants underwent walking and running sessions, each five minutes long, on a treadmill set at a constant speed of 4 and 8 km/h, respectively. Energy expenditure (EE) during walking and running was calculated using the successive 60-s interval with the lowest COV (10%) for VO_2_ and VCO_2_ throughout the final three minutes of each activity. According to the measured VO_2_ consumption (1 MET = 3.5 mL/min/kg), the mean ± SD walking intensity corresponded to 3.41 ± 0.42 MET in men and 3.54 ± 0.44 MET in women, and the mean running intensity corresponded to 8.43 ± 0.72 MET in men and 8.75 ± 0.62 MET in women. The difference in EE from RMR (∆ EE) for both walking and running exercises was calculated and used in statistical analysis as ∆ walking EE and ∆ running EE.

### 4.3. IL-8 and MCP-1 Measurement

Venous blood samples were obtained directly before and 5 min after the last bout of physical activity. At each time point, blood was drawn from the antecubital vein, blood samples were centrifuged, and serum was collected and stored at −80 °C until analysis. For the quantification of IL-8 and MCP-1 in serum, a Luminex^®^ xMAP^®^ technology (Milliplex^®^ Map Human Cytokine/Chemokine/Growth factor Panel A (Cat. No.: HCYTA-60K), intra-assay precision as % CV < 15%, EMD Millipore Corporation, Billerica, MA, USA) was used. To decrease inter-kit assay variability, the same plate was used to analyse all IL-8 and MCP-1 samples from each participant.

### 4.4. Statistical Analysis

Statistical analysis was performed using SPSS Statistics v. 26.0 (IBM Corporation, Armonk, NY, USA) with significance set at *p* < 0.05 and JMP v.13 (SAS Institute, Cary, NC, USA). The analysis included descriptive statistics, and the results were presented as means and standard deviations (SD). An independent sample t-test was used to evaluate parameter differences between sexes and groups due to changes in IL-8 and MCP-1, or the Mann–Whitney test in cases of non-Gaussian data distribution. Model-based clustering was used to group the measured multivariate data. Spearman’s test was applied to determine correlations between chemokines and anthropometric and metabolic characteristics.

## 5. Conclusions

The findings of the current study confirm that acute aerobic exercise influences the secretion of pro-inflammatory chemokines, as an increase in IL-8 and a decrease in MCP-1 were detected in the majority of participants. The observed differences in the outcomes highlight the existence of both gender and individual variations in changes in chemokine secretion as a response to the same exercise situation, and their associations with the values of metabolic parameters require further investigation.

## Figures and Tables

**Table 1:** Anthropometric characteristics of the participants.

Variable (Units)	Men Mean ± SD (*n* = 33)	Women Mean ± SD (*n* = 39)	*p* ^1^
Age (years)	31.6 ± 5.6	28.9 ± 5.0	0.033
Height (cm)	184 ± 7	170 ± 6	<0.001
Body mass (kg)	81.1 ± 9.8	64.8 ± 7.5	<0.001
Waist (cm)	85.3 ± 6.8	72.7 ± 6.6	<0.001
Hips (cm)	100.1 ± 6.4	98.3 ± 6.0	0.246
BMI (kg/m^2^)	24.1 ± 2.5	22.4 ± 2.7	0.009
Body fat (% from body mass)	15.0 ± 5.3	24.0 ± 5.3	<0.001
Lean body mass (kg)	68.7 ± 6.8	49.0 ± 4.4	<0.001
LBMI (kg/m^2^)	20.4 ± 1.4	17.0 ± 1.5	<0.001

^1 ^*p* value of sex difference due to independent samples *t*-test.

**Table 2:** Correlation of pre-exercise IL-8 and MCP-1 concentration with body parameters and RMR.

Variable (Units)	Sex	Pre-Exercise IL-8	Pre-Exercise MCP-1
Body mass (kg)	men	0.404 *	
Waist (cm)	men	0.445 **	
Lean body mass (kg)	men	0.412 *	
LBMI (kg/m^2^)	men	0.420 *	
RMR (kcal/24 h/kg)	women		0.326 *

* Spearman’s correlation coefficients, * *p* < 0.05, ** *p* < 0.01.

## Data Availability

The data presented in this study are available on request from the corresponding author.
